# Tumor-promoting properties of miR-8084 in breast cancer through enhancing proliferation, suppressing apoptosis and inducing epithelial–mesenchymal transition

**DOI:** 10.1186/s12967-018-1419-5

**Published:** 2018-02-23

**Authors:** Yujing Gao, Hongning Ma, Chanchan Gao, Ye Lv, XueHua Chen, Rongrong Xu, Miao Sun, Xinrui Liu, Xiaohong Lu, Xiuying Pei, Pu Li

**Affiliations:** 1 Key Laboratory of Fertility Preservation and Maintenance of Ministry of Education, Department of Biochemistry and Molecular Biology, School of Basic Medical Sciences, Ningxia Medical University, Yinchuan, China; 2 Department of Pediatrics, Ruijin Hospital and Ruijin Hospital North, Shanghai Jiao Tong University School of Medicine, 197 Ruijin Er Rd, Shanghai, 200025 People’s Republic of China; 3 Oncology Department of Cancer Hospital, General Hospital, Ningxia Medical University, Yinchuan, China; 4 Department of Oncology, Zhongda Hospital, Southeast University, Nanjing, China

**Keywords:** Breast cancer, miR-8084, Tumorigenesis, ING2

## Abstract

**Background:**

Breast cancer is one of the most frequent malignancies and the second leading cause of cancer-related mortality in women. MicroRNAs play a key role in breast cancer development and progression. microRNA(miR)-8084 has been observed an aberrant expression in breast cancer. However, the functions and regulatory axes of miR-8084, particularly in breast cancer, were not entirely clear.

**Methods:**

miR-8084 expression in breast cancer were investigated in a GEO dataset by in silico analysis and in 42 paired tumor tissues by qPCR. The effects of deregulation of miR-8084 on breast cancer cell proliferation, migration and invasion in vitro and tumorigenicity in vivo were examined by colony-formation assay, wound healing assay, transwell assay and nude mouse subcutaneous tumor formation model. The target gene of miR-8084 were predicted by TargetScan and miRDB, and confirmed by luciferase reporter system. The roles of miR-8084 in the breast cancer cell proliferation, apoptosis and epithelial–mesenchymal transition (EMT) were investigated by MTS, FACS and associated-marker detection by western blot.

**Results:**

miR-8084 is significantly up-regulated in both serum and malignant tissues from the source of breast cancer patients. miR-8084 promotes the proliferation of breast cancer cells by activating ERK1/2 and AKT. Meanwhile miR-8084 inhibits apoptosis by decreasing p53-BAX related pathway. miR-8084 also enhances migration and invasion by inducing EMT. Moreover, the tumor suppressor ING2 is a potential target of miR-8084, and miR-8084 regulatory axes contribute to pro-tumor effect, at least partially through regulating ING2.

**Conclusion:**

Our results strongly suggest that miR-8084 functions as an oncogene that promotes the development and progression of breast cancer, and miR-8084 is a potential new diagnostic marker and therapeutic target of breast cancer.

## Background

Breast cancer is one of the most frequent carcinoma and the second leading cause of cancer-related mortality in women, with an estimated 1.5 million new cases per year [1, 2]. The pathological progression of breast cancer is multistage and complicated, consisting of oncogenesis, proliferation, apoptosis, invasion and metastasis [3].

18–22 nucleotide-length microRNAs (miRNAs) are highly conserved small non-coding RNAs that regulate gene expression at the post-transcriptional level, and they are involved in many signaling pathways [4]. miRNAs induce mRNA degradation or translational repression by binding to seed sequences in the 3′-untranslated regions (3′-UTRs) of their targets [5]. Many studies have reported deregulation of microRNAs expression profiling between tumor cells and cells derived from normal tissues, indicating that miRNAs and potential altered gene may contribute to tumorigenicity in various cancers [6–8]. Deregulation of microRNAs has been described as a determinant for the initiation and progression of carcinomas, including breast cancer [9–11]. Many studies have confirmed the key roles of miRNAs in breast cancer. miRNAs have been linked to all stages in breast cancer [12, 13].

In our previous work, we constructed a pair of breast cancer cell lines with different tumorigenesis and migration ability and the miRNAs profiles of them were detected by microarray assay. miR-8084 is one of the most significant up-regulated miRNAs in the cells with higher ability of tumorigenesis and migration, indicating miR-8084 may function as an oncogene in breast cancer.

miR-8084 is a relatively new recognized miRNA. The functional annotation and the expression pattern of miR-8084 have not been described clearly. So far, there are only two studies which reported the deregulation of miR-8084 expression in osteoporosis [14] and epithelial ovarian tumor [15] respectively. The function of miR-8084 in breast cancer is completely unknown. By analyzing a newly published GEO data, we found that miR-8084 is one of the miRNAs which up-regulated in serum from the source of breast cancer patients [16]. This result is consistent with our pervious data in breast cancer cells. It gave us a clue that miR-8084 may play an important role in tumorigenesis and progression of breast cancer. Due to its detectability in the serum, miR-8084 is expected to be a new diagnosis biomarker for breast cancer.

In this study, we are investigating the function and molecular mechanism of miR-8084 in breast cancer and exploring the practicability of using miR-8084 as a potential diagnostic marker and therapeutic target.

## Methods

### Cell culture

Human breast cancer cell lines, MDA-MB-231 and BT-549, were purchased from American Type Culture Collection (ATCC). Both cells were cultured in RPMI-1640 Medium (Gibco, USA) with 10% fetal bovine serum (FBS) (Hyclone, USA). Cells were maintained at 37 °C, 5% CO_2_ in a humidified incubator.

### RNA isolation and qRT-PCR

Total RNA was extracted from tissues using a mirVana™ miRNA Isolation Kit (Applied Biosystems, Foster City, CA, USA) according to the manufacturer’s instructions. Concentrations and purity of the RNA samples were measured by electrophoresis and spectrophotometric methods. The expression level of miR-8084 in tissues was assayed by qRT-PCR and calculated.

### Transient transfection

miR-8084 mimics, negative control mimics, miR-8084 inhibitors and inhibitor control were purchased from RiboBio Biotechnology Co. Ltd. (Guangzhou, China). Cells were seeded into cell culture plates 20 h before transfection to ensure 70% cell confluence at the moment of transfection. Transfection of oligonucleotides into breast cancer cells was carried out using Lipofectamine2000 (Invitrogen, Carlsbad, CA, USA) according to the manufacturer’s procedure.

### Cell proliferation assay

Triplicates of 1 × 10^3^ cells in the 100 μl medium were plated into each well of the 96-well plates. At 24, 48, 72, and 96 h, cell proliferation was assessed by Cell Titer 96^®^ Aqueous One Solution Cell Proliferation Assay (Promega, Madison, WI, USA) according to the protocol provided by the manufacturer. Briefly, 20 μl of MTS/PMS solution was added to each well and incubated for another 1–4 h at 37 °C in a humidified condition of 5% CO_2_. The absorbance at 490 nm was read and recorded using an ELISA plate reader (Model 680, Bio-Rad Laboratories Inc., Hercules, CA, USA).

### Wound healing assay

Wound healing assay was used to detect the capacity for cells motility. For scratch wound healing assay, cells were cultured in serum-free medium for 24 h and wounded with pipette tips. Fresh medium was replaced. The wound closing procedure was observed for 24 h and photographs were taken.

### Colony formation assay

In plate colony formation assay, cells were resuspended in RPMI 1640 containing 10% FBS and layered onto six-well plates (1 × 10^3^ cells/well). The cells were incubated for 2 weeks and stained with crystal violet. Colonies containing 50 cells or more were counted.

### Cell migration and invasion assay

Cell migration and invasion assay were performed using transwell chamber (8 μm, 24-well insert; Corning, Lowell, MA, USA). In migration assay, Cells (3 × 10^4^) in serum-free medium were added to the upper chamber, and medium containing 10% FBS were added to the lower. Cells were then incubated for 24 h. For invasion assay, diluted Matrigel (BD Biosciences) was used to coat the insert chambers’ membrane. Cells were cultured for 48 h under the same conditions. Finally, cells that migrated or invaded into the lower chambers were fixed with methanol, stained with crystal violet and counted in six random fields.

### In vivo xenotransplanted tumor model

SPF grade female BALB/c nude mice were purchased from Institute of Zoology, Chinese Academy of Sciences. BT-549 cells with stable overexpression of miR-8084 were obtained by leti-virus infection and subsequent puromycin selection. BT-549 cells with or without miR-8084 overexpression were resuspended in PBS and subcutaneously injected into 4-week-old female nude mice, respectively (5 × 10^6^ cells/0.2 ml PBS per mouse, 5 mice in each group). The length (L) and width (W) of each formed tumor were measured every 5 days with calipers, and the volume was calculated using the formula: (W + L)/2 × W×L × 0.5236. The mice were euthanized 35 days after the injection, and the tumors were weighed.

### Luciferase reporter activity assay

The 293T cells were cultured in 24-well plates and transfected with 0.2 μg luciferase reporter plasmid pmir-GLO (Promega, Madison, WI, USA) containing either wide-type or mutant 3′UTR of ING2, together with 1 μg oligonucleotides. Transfection was performed using Lipofectamine2000 reagent (Invitrogen). Relative luciferase activity was calculated 36 h post-transfection by the Dual Luciferase Reporter Assay (Promega).

### Statistical analysis

The difference between two groups were analyzed using Student *t* test. All statistical analyses were performed using the SPSS 17.0 software (SPSS Inc, Chicago, IL, USA), and *P* < 0.05 was considered as significant.

## Results

### miR-8084 is up-regulated in breast cancer

**Fig. 1:**
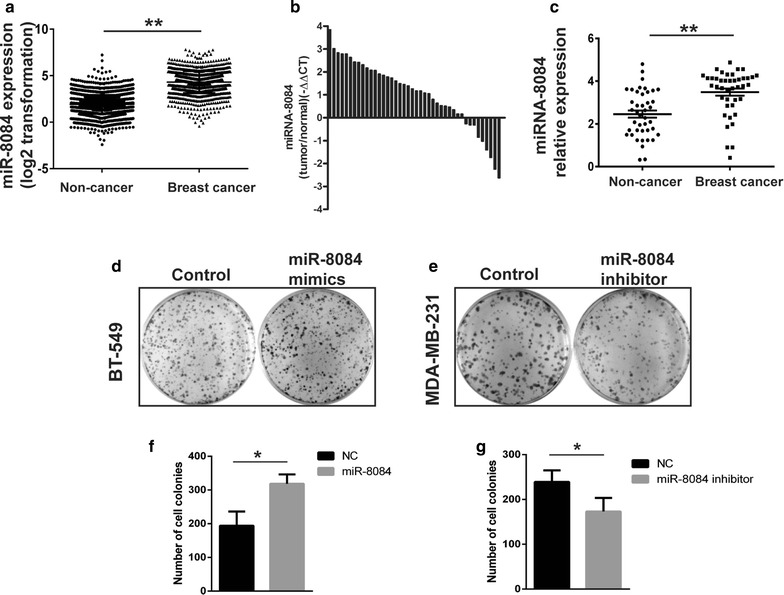
miR-8084 is up-regulated in breast cancer and promotes the clonogenicity of breast cancer cells. **a** miR-8084 in serum of breast cancer and that of non-cancer samples was analyzed using data from GEO (GSE73002) (**P < 0.01). **b** miR-8084 was detected in breast cancer tissues. The data is shown as ^−△△^CT values. **c** Relative expression of miR-8084 in breast cancer tissues compared with normal tissues. Data is shown as Mean ± SEM (**P < 0.01). **d** The effect of miR-8084 on clonogenicity was evaluated by the plate colony formation assay. BT-549 cells were transfected with miR-8084 mimics or control, seeded onto six-well plates. The number of colonies was counted on the 14th day after seeding. **e** MDA-MB-231 cells were transfected with miR-8084 inhibitor or control then seeded onto six-well plates. The number of colonies was counted on the 14th day after seeding. **f**, **g** Colonies containing 50 or more cells were counted. Results are means of three independent experiments ± SD (*P < 0.05)

In our previous work, we found that miR-8084 was up-regulated in breast cancer cell line with higher ability of proliferation and migration. Furthermore, we compared the levels of miR-8084 in serum of breast cancer and that of non-cancer samples using data from GEO (GSE73002), which including 1280 cases of breast cancer and 2686 cases of non-cancer control. As shown in Fig. 1a, miR-8084 level in breast cancer is significantly higher than that in non-cancer samples (**P < 0.01).

We next examined the expression levels of miR-8084 in 42 pairs of breast cancer tissues and matched adjacent normal tissues by qRT-PCR. The results suggested that increasing of miR-8084 was a frequent event in breast cancer, approximately 79% (33/42 samples, Fig. 1b), and the average relative expression level of miR-8084 was significantly up-regulated in tumor tissues compared to the non-tumor tissues (Fig. 1c, **P < 0.01).

### miR-8084 promotes clonogenicity of breast cancer cells in vitro

Considering that miR-8084 is significantly up-regulated in breast cancer, it may function as an oncogene. However, the functions and molecular mechanisms have not been investigated. Clonogenicity is one of the basic aspects of tumorigenicity, therefore we tested whether deregulation of miR-8084 in breast cancer cells could affect cell clonogenicity. First, we examined the expression level of miR-8084 in different breast cancer cell lines, and chose MDA-MB-231 and BT-549 cells which contain relatively high- and low-level of miR-8084 respectively to do the following experiments. The plate colony formation assay was performed after miR-8084 mimics or anti-sense inhibitor and the corresponding negative control oligonucleotides were transfected into breast cancer cells (BT-549 or MDA-MB-231) respectively. The results showed that the number of colonies from breast cancer cells transfected with miR-8084 mimics was significantly much more than that from the control group (BT-549^miR-8084^ versus BT-549^NC mimics^, *P < 0.05; (Fig. 1d, f), while the colony number from cells transfected with miR-8084 inhibitor was fewer when compared with negative control group. (MDA-MB-231^miR-8084 inhibitor^ versus MDA-MB-231^NC inhibitor^, *P* < 0.05, Fig. 1e, g). These findings indicate that miR-8084 promotes colony forming ability of breast cancer cells.

### miR-8084 promotes migration and invasion of breast cancer cells in vitro

**Fig. 2:**
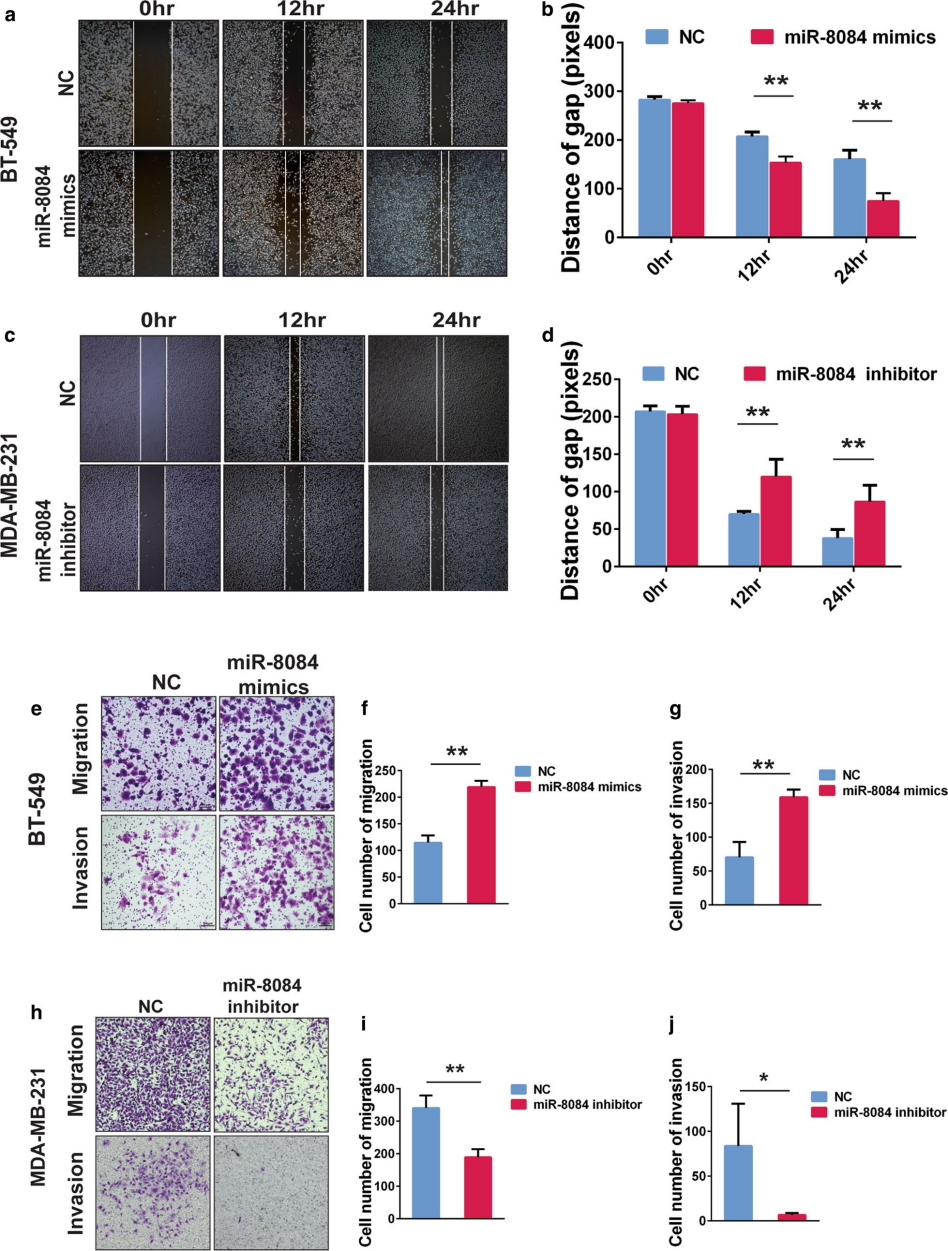
miR-8084 enhances scratch wound healing ability, migration and invasion of breast cancer cells. **a** Movement ability of BT-549^NC^, BT-549^miR-8084^ cell lines was detected by scratch wound healing assays. **b** Cell migration was quantified as distance of wound-healed area. Data represent mean ± SD (**P < 0.01). **c** Movement ability of MDA-MB-231^NC^ or MDA-MB-231^miR-8084 inhibitor^ cell lines was detected by scratch wound healing assays. **d** Cell migration was quantified as distance of wound-healed area. Data represent mean ± SD (**P < 0.01). **e** Representative photographs of migratory or invasive BT-549^NC^ or BT-549^miR-8084^ cells on the membrane (magnification, ×100). **f**, **g** Average number of migratory or invasive BT-549^NC^ or BT-549^miR-8084^ cells. (**P < 0.01). **h** Representative photographs of migratory or invasive MDA-MB-231^NC^ or MDA-MB231^miR-8084 inhibitor^ cells on the membrane (magnification, ×100). **i**, **j** Average number of migratory or invasive MDA-MB-231^NC^ or MDA-MB231^miR-8084 inhibitor^ cells (*P < 0.05, **P < 0.01). The data represent the mean ± SD

In order to further assess the influence of miR-8084 on breast cancer cells, we investigated its effects on cell migration and invasion, a characteristic ability of tumor progression and metastasis. Movement ability is the basic property of migration and invasion. The horizontal cellular movement ability of breast cancer cells was assessed by wound healing assay. As shown in Fig. 2, miR-8084 ectopic-expression cell line BT-549^miR-8084^ sealed the wound more efficient than the control BT-549^NC mimics^ cell line (Fig. 2a, b). In contrast, the MDA-MB-231^miR-8084 inhibitor^ cells sealed the scratch wounds more slowly than that of the control MDA-MB-231^NC inhibitor^ cell line (Fig. 2c, d). We then used transwell assay to evaluate the effects of miR-8084 on vertical cellular migration and invasion. The results showed that ectopic expression of miR-8084 led to the migratory and invasive ability of breast cancer cells enhanced significantly and vice verse as shown in Fig. 2e, h. The number of migratory BT-549 cells transfected with miR-8084 mimics was significantly more than the control group, whereas the number of MDA-MB-231 cells transfected with miR-8084 inhibitor was less than the control group (**P < 0.01, Fig. 2f, i); the results of invasive ability performance groups are also consistent. (*P < 0.05, **P < 0.01, Fig. 2g, j). Our data clued that miR-8084 is an important participant in the migration and invasion ability of breast cancer cells.

### miR-8084 contributes to tumorigenicity of breast cancer cells in vivo

**Fig. 3:**
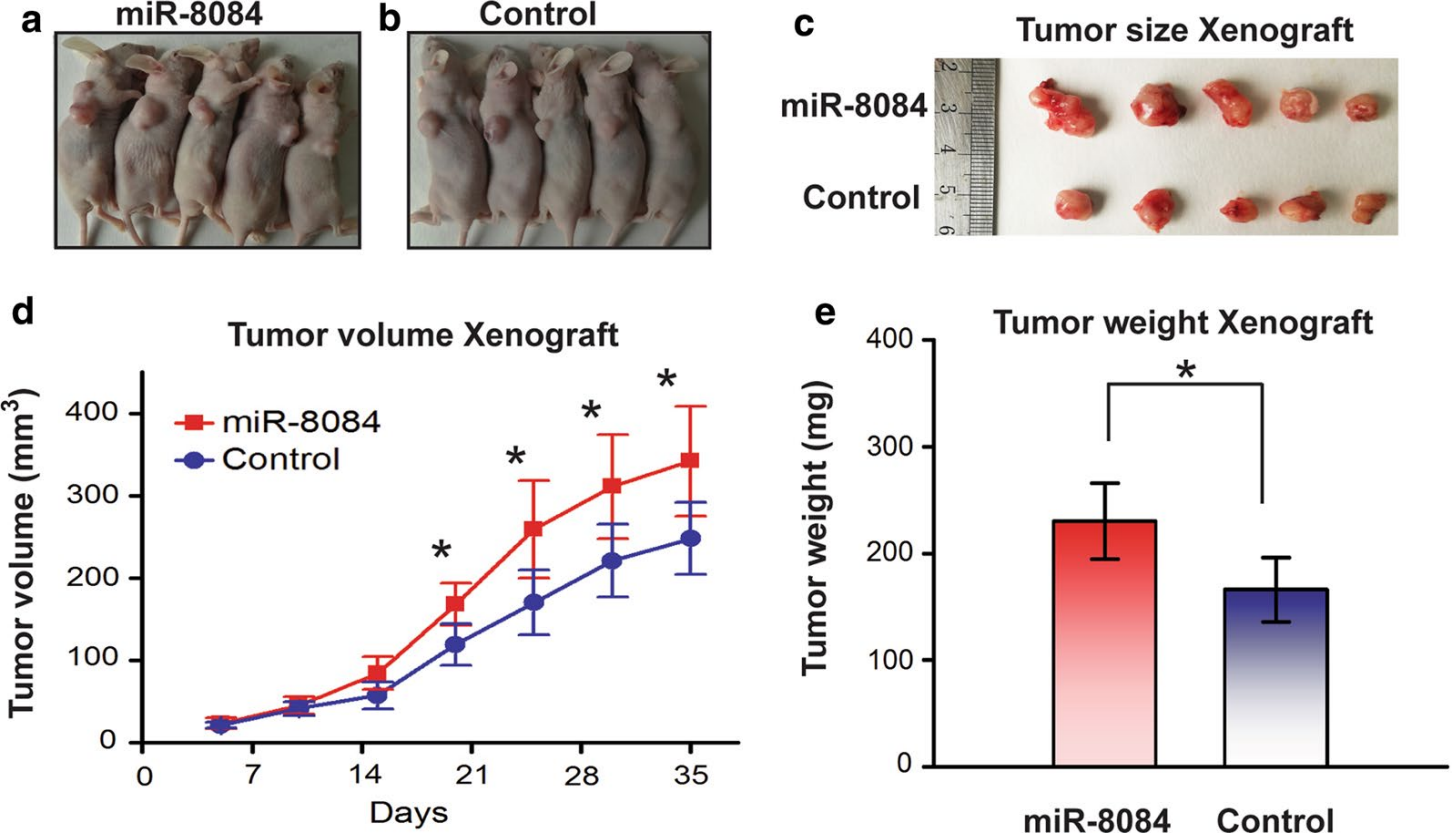
miR-8084 contributes to tumorigenicity of breast cancer cells in vivo. **a**–**c** Photographs of tumors derived for miR-8084 or control stably transfected BT-549 cells in nude mice. **d** Growth kinetics of tumors in nude mice. Tumor diameters were measured every 5 days (*P < 0.05). **e** Average weight of tumors in nude mice. Mean ± SD were shown (*P < 0.05)

We proceeded to validate whether ectopic expression of miR-8084 could affect the tumorigenicity of breast cancer cells in vivo. To explore the contribution of miR-8084 in vivo, mouse xenograft models were carried out. BT-549 cells with or without ectopic expression of miR-8084 were injected subcutaneously into female nude mice, and the tumor formation was monitored. Consistent with the results from in vitro experiments, the tumor grew progressively in the miR-8084 ectopic expression group compared to the control group (Fig. 3a–c). The diameters of the tumors were measured and the volumes were calculated every 5 days. These mice were euthanized 35 days after the injection, and the tumors were weighed (Fig. 3d, e). Obviously, the tumor volumes and weights of the BT-549^miR-8084^ group were significantly higher than data of the control group. Thus, these results indicate that miR-8084 could promote tumorigenesis of breast cancer cells in vivo.

### miR-8084 targets the 3′-UTR of *ING2*

**Fig. 4:**
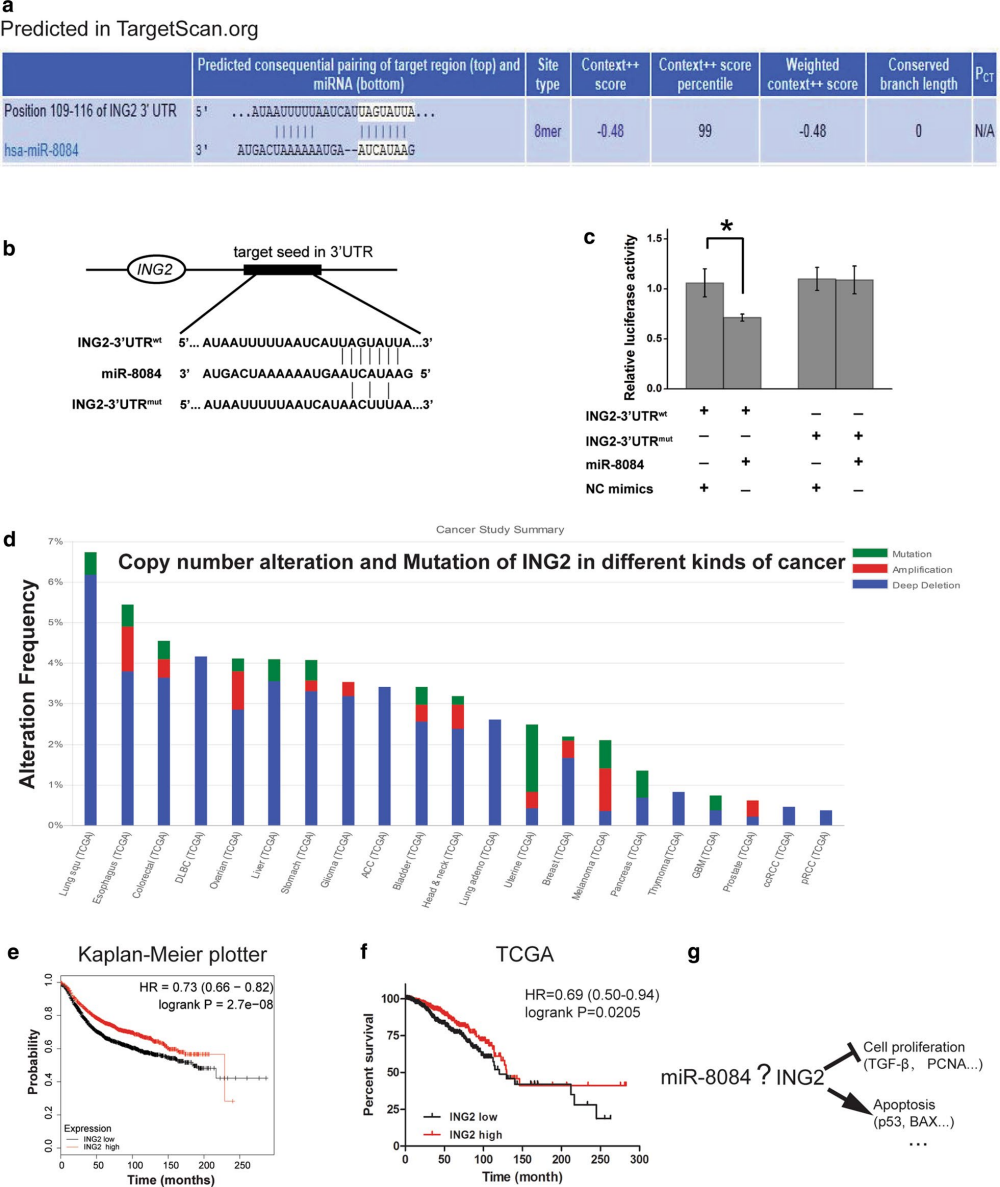
miR-8084 targets the 3′-UTR of *ING2*. **a** The putative binding sites of miR-8084 in *ING2* 3′-UTR region were predicted. The matched seed sequences were indicated by vertical lines. **b** Schematic graph of the putative binding sites of miR-8084 in the *ING2* 3′UTR and the mutation in miR-8084 binding sites. **c** miR-8084 mimics down-regulated luciferase activities controlled by wild-type *ING2* 3′UTR, but did not affect luciferase activity controlled by mutant *ING2* 3′UTR. The results are means of three independent experiments ± SD (*P < 0.05). **d** The Cancer Gemone Atlas online database was queried using cBioportal to analysis ING2 gene alteration in human cancers. **e** The survival curves was performed by Kaplan–Meier plotter (http://kmplot.com/analysis/; probe ID: 213544_at). Totally, 3951 patients were included, 1977 cases were in ING2 low-expression cohort, and 1974 cases were in ING2 high-expression cohort. The median survival for ING2 high-expression cohort is 228.85 months, and 185.16 months for ING2 low-expression cohort. **f** Survival analysis for breast cancer using data from TCGA. 1091 patients were included, 546 cases were in ING2 low-expression cohort, and 545 cases were in ING2 high-expression cohort. The median survival for ING2 high-expression cohort is 129.6 months, and 120.53 months for ING2 low-expression cohort. 449 and 478 cases are censored in ING2 low-expression and high-expression cohort respectively. **g** ING2 participates in the regulation of genes involved in cell proliferation, cell cycle, and apoptosis

**Fig. 5:**
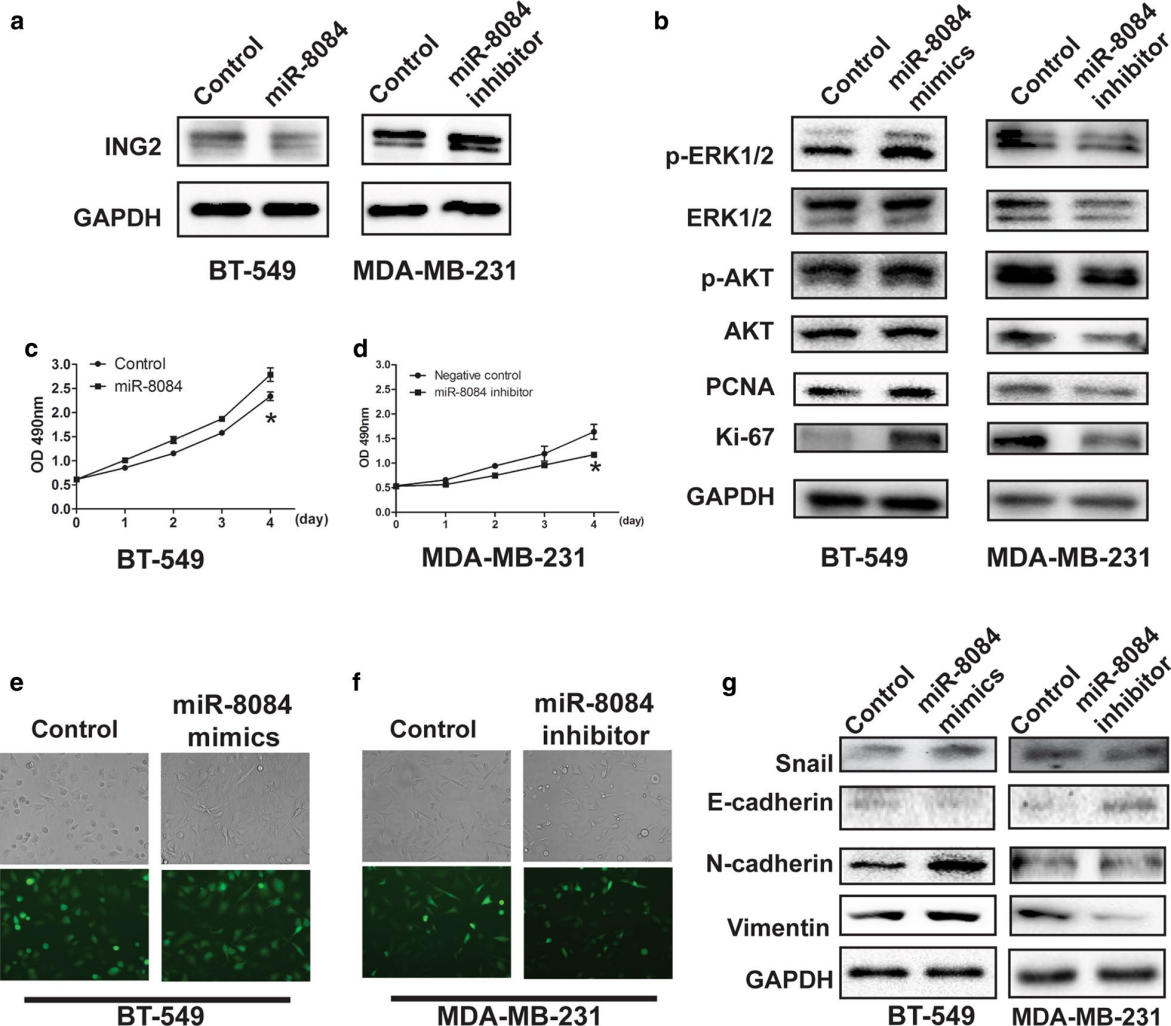
miR-8084 enhances the proliferation and epithelial-to-mesenchymal transition. **a** Protein levels of ING2 in BT-549^NC,^ BT-549^miR-8084^, MDA-MB-231^NC^ and MDA-MB-231^miR-8084 inhibitor^ cells were detected by western blotting. **b** Relative protein levels of proliferation related markers (AKT, ERK1/2, PCNA and Ki-67) in BT-549^NC,^ BT-549^miR-8084^, MDA-MB-231^NC^ and MDA-MB-231^miR-8084 inhibitor^ cells were analyzed by western blotting. **c** Cell proliferation was measured by the MTS assay. BT-549 cells were transfected with miR-8084 mimics or control, at 24 h post-transfection, the MTS assay was performed every 24 h for 4 days (*P < 0.05). **d** MDA-MB-231 cells were transfected with miR-8084 inhibitor or control, at 24 h post-transfection, the MTS assay was performed every 24 h for 4 days (*P < 0.05). **e** EMT-like morphology of BT-549 cells transfected with miR-8084 mimics or control. **f** EMT-like morphology of MDA-MB-231 cells transfected with miR-8084 inhibitor or control. **g** Relative protein levels of EMT markers (epithelial cell marker E-cadherin and the mesenchymal cell markers N-cadherin, vimentin and snail) in BT-549^NC,^ BT-549^miR-8084^, MDA-MB-231^NC^ and MDA-MB-231^miR-8084 inhibitor^ cells were analyzed by western blotting

To identify the potential target of miR-8084, computational prediction was performed using bioinformatics tools (e.g. TargetScan and miRDB) [17, 18]. Among all the hits, ING2 (inhibitor of growth family member 2) caught our attention (Fig. 4a). ING2 is a characterized tumor suppressor which plays an anti-tumor role by affecting cell proliferation, apoptosis and migration. Then we performed luciferase reporter assays to verify a direct interaction between miR-8084 and the 3′UTR of *ING2*. Luciferase reporters were constructed by inserting either a wild-type *ING2* 3′UTR sequence containing the seed sequence for miR-8084 recognition (ING2-3′UTR^wt^), or a mutated 3′UTR (ING2-3′UTR^mut^) (Fig. 4b). The relative luciferase activity of the *ING2*-3′UTR^wt^ reporter was significantly suppressed by miR-8084 mimics compared to the NC mimics; however, once the seed sequence for miR-8084 was mutated, this inhibitory effect was disappeared (Fig. 4c). We further confirmed miR-8084 mimics could decrease the protein level of ING2, while inhibitors for miR-8084 could elevate the protein level of ING2 (Fig. 5a). These results strongly indicate that 3′UTR of *ING2* carries the direct binding seed of miR-8084, and miR-8084 might target *ING2* and function as an oncogene.

When we analyzed the copy number alteration and mutation of ING2 in different kinds of cancer using data from TCGA (http://cbioportal.org), we found the most frequent gene alteration for ING2 is deep deletion (Fig. 4d), which is consistent with the function as a tumor suppressor. Furthermore, the results of survival analysis for breast cancer indicate patients with high expression of ING2 have higher probability of long-term survival (http://kmplot.com/analysis/, http://cbioportal.org; Fig. 4e, f). Furthermore, ING2 is also a favorable prognostic factor for survival in different subtype of breast cancer (including Basal, luminal A, luminal B and HER2-positive). (http://kmplot.com/analysis/).

### miR-8084 enhances the proliferation of breast cancer cells by activating ERK1/2 and AKT

ING2 has been reported to participate in the regulation of genes involved in cell proliferation, cell cycle and apoptosis (Fig. 4g). As a potential regulator of ING2, it is reasoned that ectopic expression of miR-8084 could induce changes of biological phenotypes by reducing ING2 expression in breast cancer. Given the reported inhibitory effect of ING2 on cell proliferation, we hypothesized that miR-8084 may promote cell proliferation via the related signaling pathways. To verify this, proliferation of breast cancer cells was monitored after transfected with miR-8084 mimics or inhibitors. The results showed that miR-8084 mimics could promote proliferation of BT-549 cells, while miR-8084 inhibitors could suppress that of MDA-MB-231 cells (Fig. 5c, d). ERK1/2 and AKT were activated by miR-8084 mimics; protein levels of proliferation related markers Ki-67 and proliferating cell nuclear antigen (PCNA) were elevated by miR-8084 mimics. Conversely, inhibitors for miR-8084 suppress the activation of ERK1/2 and AKT, as well as the expression of Ki-67 and PCNA (Fig. 5b). Thus the data provided evidence that miR-8084 promotes proliferation by activating ERK1/2 and AKT, which is due to suppression of ING2, at least partially.

### miR-8084 induces epithelial-to-mesenchymal transition of breast cancer cells

Since epithelial to mesenchymal transition (EMT) confers metastatic potential of cancer cells [32], we postulated that miR-8084 might enhance migration and invasion by inducing EMT of breast cancer cells. To prove this postulation, breast cancer cells were transfected with either miR-8084 mimics or miR-8084 inhibitor, and the expression of the epithelial cell marker E-cadherin and the mesenchymal cell markers N-cadherin, vimentin and snail was analyzed. As shown in Fig. 5e, miR-8084 mimics induced an EMT-like morphology of BT549 cells, which exhibited a spindle-like and fibroblastic appearance; and the expression of mesenchymal markers was up-regulated and the expression of epithelial cell marker was down-regulated (Fig. 5g). In contrast, miR-8084 inhibitors inhibited EMT of MDA-MB-231 cells according to morphological change of cells (Fig. 5f) and protein levels of mesenchymal markers (Fig. 5g).

### miR-8084 inhibits apoptosis by decreasing p53-BAX related pathway

**Fig. 6:**
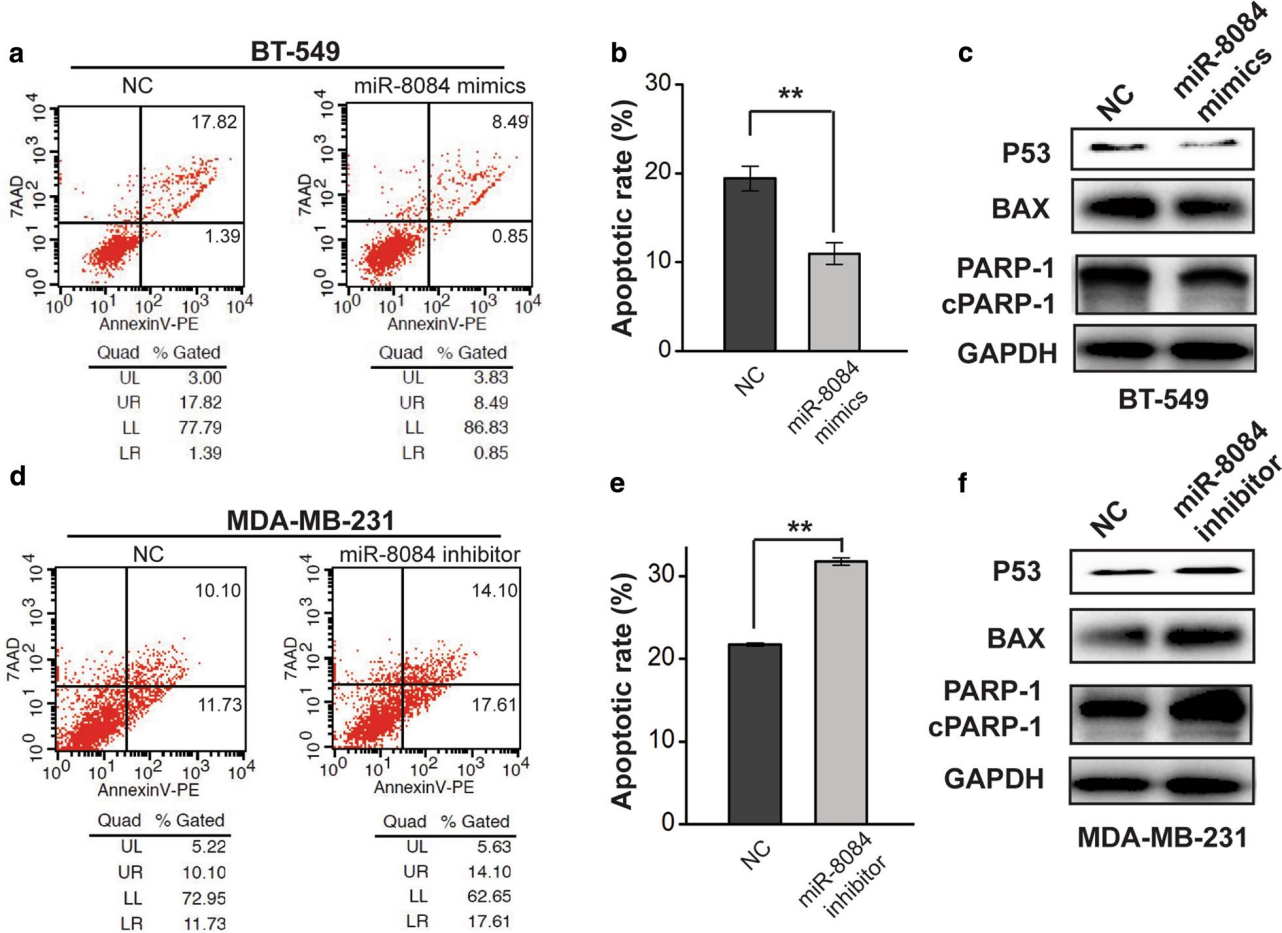
miR-8084 inhibited apoptosis by decreasing p53-BAX related pathway. **a** Representative histograms depicting apoptosis of BT-549 transfected with miR-8084 mimics or control. **b** The percentage of apoptotic cells of three independent experiments ± SD (**P < 0.01). **c** Relative levels of apoptosis related markers (p53, BAX and PARP-1) in BT-549^NC^ or BT-549^miR-8084^. **d** Representative histograms depicting apoptosis of MDA-MB-231 transfected with miR-8084 inhibitor or control. **e** The percentage of apoptotic cells of three independent experiments ± SD (**P < 0.01). **f** Relative levels of apoptosis related markers (p53, BAX and PARP-1) in MDA-MB-231^NC^ or MDA-MB-231^miR-8084 inhibitor^

Anti-apoptotic ability is an important characteristic of tumor cells. Previous study reported that ING2 induced apoptosis via p53 related pathway [19]. As a negative regulator for ING2, miR-8084 might hold an opposite effect to ING2 on apoptosis. We then analyzed the role of miR-8084 in serum starvation-induced apoptosis by flow cytometry. The results indicated that the apoptotic rate was significantly decreased in miR-8084 mimics transfected BT-549 cells compared to the control cells (**P < 0.01; Fig. 6a, b). Consistently, inhibition of miR-8084 enhanced apoptosis of MDA-MB-231 cells (**P < 0.01; Fig. 6d, e). Considering that ING2 can inhibit apoptosis by regulating p53 and BAX, we tested whether miR-8084 could influence the expression level of these apoptosis-related genes. As shown in Fig. 6c, f, ectopic expression of miR-8084 inhibited the expression levels of BAX and p53, as well as the expression and activation of PARP-1, in contrast, inhibition of miR-8084 enhanced the levels or activation of those proteins. These results indicated that miR-8084 could inhibit apoptosis in breast cancer cells, at least partly by regulating p53-BAX pathway.

## Discussion

Recent years, an increasing number of evidences have supported that miRNAs hold very important roles in many biological process, it would never be considered to be “Junk” as at the initial stage [20]. In the oncology research, miRNAs widely participate in tumorigenesis and progression of all kinds of tumors. Due to the detectability in serum, miRNAs can be used as diagnostic and prognostic biomarkers for cancers. miR-8084 is a novel recognized miRNA with few studies reported its function in disease. Here, we investigate the role of miR-8084 in breast cancer for the first time. We found miR-8084 is significantly up-regulated in both serum and tissue samples from source of breast cancer patients. This result supported that miR-8084 is expected to be a novel diagnostic marker for breast cancer. However, further large-sample investigation is needed to verify this result.

miRNAs promoting oncogenesis, progression and metastasis are classified as “oncomiRs” [21–23]. OncomiRs performed their oncogenic activity by suppressing expression of tumor suppressor genes through either decreasing translation or increasing degradation of targeted mRNA [24, 25]. Our results indicate miR-8084 functions as an oncomiR which is able to suppress apoptosis and promote cell growth, cell migration and invasion in breast cancer; and the target gene for miR-8084 to carry out its pro-tumor function is ING2, which is the first reported potential target of miR-8084.

ING2, a member of the ING protein family, contains a highly conserved plant homeodomain (PHD) and nuclear localization sequences (NLS) [26, 27]. It has been considered as a tumor suppressor gene. Expression of ING2 is decreased in several kinds of cancer including lung cancer [28, 29], hepatocellular carcinoma [29] and breast cancer [30]. ING2 can bind to the histone H3 trimethylated at lysine4 (H3K4me3), indicating that it has potential ability of transcriptional transactivation [31–33]. ING2 participates in the regulation of genes in several signal pathways, including p53-BAX, TGF-β, and PCNA. As a negative regulator of ING2, miR-8084 could be involved in the aforementioned signaling pathways through targeting ING2.

Proliferation is a key property of cancers, which is widely estimated by the assessment of factors like PCNA or Ki-67. PCNA, a nuclear protein, is essential for DNA replication and repair. Inhibition of PCNA could reduce AKT phosphorylation [34]. AKT, an oncogenic protein, promotes proliferation and reduces apoptosis in breast cancer [35]. The activation of AKT/ERK signaling is a key sign of promotion of proliferation, which is evaluated by their phosphorylated form. ING2 interacts with PCNA through its NCR domain, regulating its post-translational modification and DNA replication [36, 37]. ING2 could regulate AKT/ERK signals via PCNA. Our results showed that miR-8084 could enhance the proliferation by increasing the expression of PCNA and Ki-67, and activating ERK1/2 and AKT.

EMT is a critical event in progression of cancer metastasis, particularly during migration and invasion when malignant tumor cells migrate to distant organs to form metastases [7, 38] EMT is characterized by decreasing of epithelial differentiation markers including E-cadherin and increasing of mesenchymal markers such as N-cadherin and vimentin. miRNAs have been shown to be crucial for EMT in the breast cancer, such as miR-200 family members [39]. TGF-β is a principal cytokine inducing EMT in breast cancer [40]. Futhermore, the potential target of miR-8084, ING2, mediates transforming growth factor-β (TGF-β) signaling [41]. Here, our results reveal that miR-8084 could promote EMT-associated migration in breast cancer; and ING2 may be a key mediator of this process.

Malignant carcinoma usually possesses the ability to avoid apoptosis, which leads to tumorigenesis and therapeutic resistance. p53 is the most important determinant of apoptosis. p53 mediates the transcriptional activation of pro-apoptotic protein such as BAX [42]. Previous study reported that ING2 deficiency induced both p53-dependent and independent apoptosis [43]. In addition, ING2 could negatively regulate cell proliferation by activating p53 [44]. Our results indicate that miR-8084 could inhibit apoptosis in breast cancer cells by suppressing p53-BAX pathway.

**Fig. 7:**
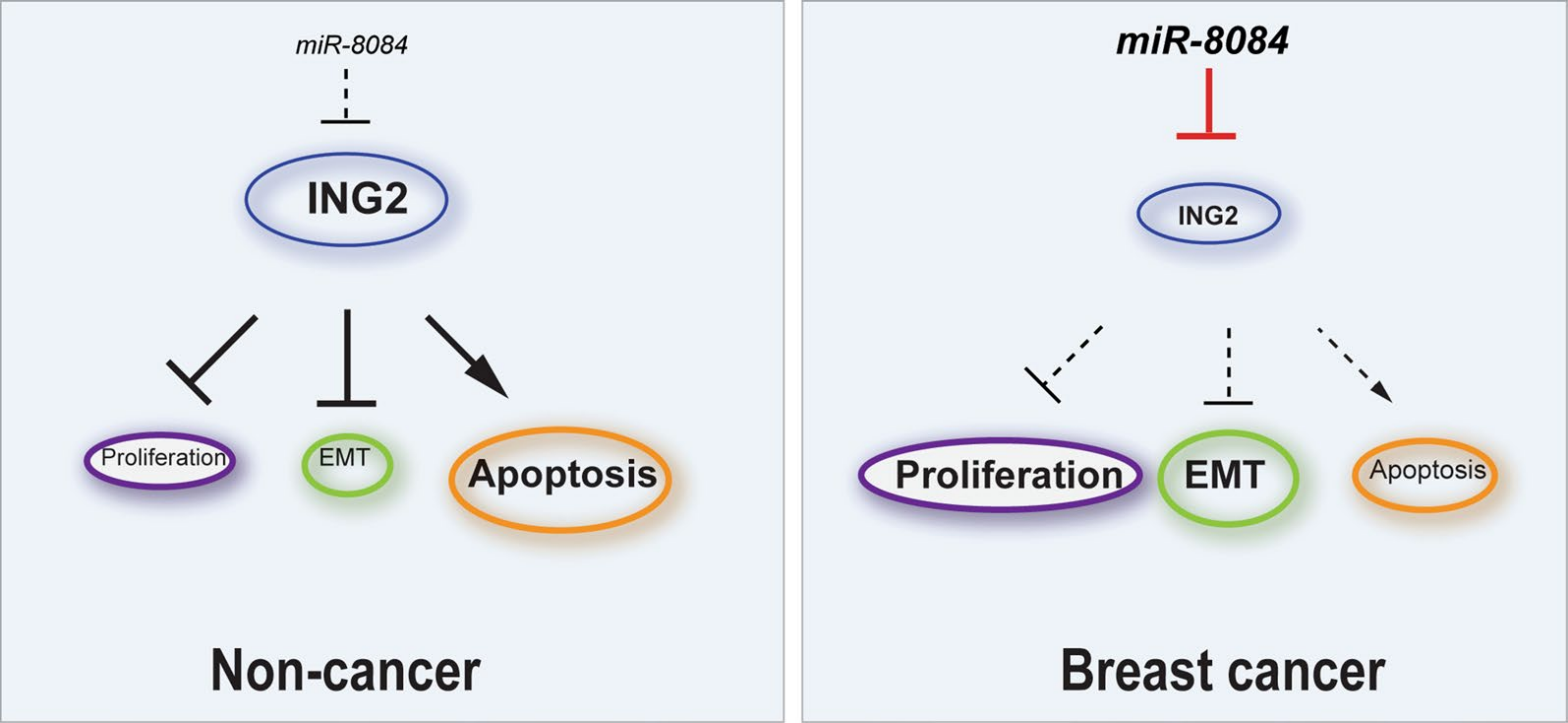
Schematic graph depicting the function of miR-8084 in breast cancer. miR-8084 enhances proliferation and epithelial–mesenchymal transition, meanwhile inhibits apoptosis in breast cancer. The increasing of miR-8084 reinforces suppression of its target *ING2*, leading to promote the tumorigenicity in breast cancer

In this work we endeavor to elucidate the role of miR-8084 in breast cancer. All these findings suggest: miR-8084 is significantly elevated in both serum and malignant tissues from the source of breast cancer patients. It functions as an oncogene that is able to suppress apoptosis and promote cell growth, cell migration and invasion, and tumorgenicity in breast cancer. The tumor suppressor ING2 is the first confirmed target of miR-8084, and miR-8084 contributes to pro-tumor effect, at least partially, through regulating ING2 (Fig. 7).

## Conclusion

Our current work is the first attempt to evaluate the pro-tumor effects of miR-8084 in breast cancer. The results strongly suggest that miR-8084 functions as an oncogene that promotes the development and progression of breast cancer and miR-8084 is a potential new diagnostic marker and therapeutic target of breast cancer.

## Abbreviations

miRNA: microRNA
EMT: epithelial–mesenchymal transition
ING2: inhibitor of growth family member 2
3′-UTRs: 3′-untranslated regions
FACS: fluorescence activated cell sorter
PCNA: proliferating cell nuclear antigen
TGF-β: transforming growth factor-β
BAX: BCL2 associated X, apoptosis regulator

## References

[1] Bombonati A, Sgroi DC (2011). The molecular pathology of breast cancer progression. J Pathol.

[2] Siegel RL, Miller KD, Jemal A (2015). Cancer statistics, 2015. CA Cancer J Clin.

[3] Zhang K, Zhang Y, Liu C, Xiong Y, Zhang J (2014). MicroRNAs in the diagnosis and prognosis of breast cancer and their therapeutic potential (review). Int J Oncol.

[4] O’Hara SP, Mott JL, Splinter PL, Gores GJ, LaRusso NF (2009). MicroRNAs: key modulators of posttranscriptional gene expression. Gastroenterology.

[5] Bartel DP (2004). MicroRNAs: genomics, biogenesis, mechanism, and function. Cell.

[6] Brueckner B, Stresemann C, Kuner R, Mund C, Musch T, Meister M, Sultmann H, Lyko F (2007). The human let-7a-3 locus contains an epigenetically regulated microRNA gene with oncogenic function. Cancer Res.

[7] Gupta GP, Massague J (2006). Cancer metastasis: building a framework. Cell.

[8] Esquela-Kerscher A, Slack FJ (2006). Oncomirs—microRNAs with a role in cancer. Nat Rev Cancer.

[9] van Schooneveld E, Wildiers H, Vergote I, Vermeulen PB, Dirix LY, Van Laere SJ (2015). Dysregulation of microRNAs in breast cancer and their potential role as prognostic and predictive biomarkers in patient management. Breast Cancer Res.

[10] Pillai RS (2005). MicroRNA function: multiple mechanisms for a tiny RNA?. RNA.

[11] Calin GA, Croce CM (2006). MicroRNA signatures in human cancers. Nat Rev Cancer.

[12] Yahya SM, Elsayed GH (2015). A summary for molecular regulations of miRNAs in breast cancer. Clin Biochem.

[13] Dykxhoorn DM (2010). MicroRNAs and metastasis: little RNAs go a long way. Cancer Res.

[14] Jiménez-Ortega RF, Ramírez-Salazar EG, Parra-Torres AY, Muñoz-Montero SA, Rangel-Escareňo C, Salido-Guadarrama I, Rodriguez-Dorantes M, Quiterio M, Salmerón J, Velázquez-Cruz R (2017). Identification of microRNAs in human circulating monocytes of postmenopausal osteoporotic Mexican-Mestizo women: a pilot study. Exp Ther Med.

[15] Chong GO, Jeon HS, Han HS, Son JW, Lee YH, Hong DG, Lee YS, Cho YL (2015). Differential microRNA expression profiles in primary and recurrent epithelial ovarian cancer. Anticancer Res.

[16] Shimomura A, Shiino S, Kawauchi J, Takizawa S, Sakamoto H, Matsuzaki J, Ono M, Takeshita F, Niida S, Shimizu C (2016). Novel combination of serum microRNA for detecting breast cancer in the early stage. Cancer Sci.

[17] Lewis BP, Burge CB, Bartel DP (2005). Conserved seed pairing, often flanked by adenosines, indicates that thousands of human genes are microRNA targets. Cell.

[18] Wong N, Wang X (2015). miRDB: an online resource for microRNA target prediction and functional annotations. Nucleic Acids Res.

[19] Pedeux R, Sengupta S, Shen JC, Demidov ON, Saito S, Onogi H, Kumamoto K, Wincovitch S, Garfield SH, McMenamin M (2005). ING2 regulates the onset of replicative senescence by induction of p300-dependent p53 acetylation. Mol Cell Biol.

[20] Berg P (2006). Origins of the human genome project: why sequence the human genome when 96% of it is junk?. Am J Hum Genet.

[21] Tang J, Ahmad A, Sarkar FH (2012). The role of microRNAs in breast cancer migration, invasion and metastasis. Int J Mol Sci.

[22] Chen L, Bourguignon LY (2014). Hyaluronan-CD44 interaction promotes c-Jun signaling and miRNA21 expression leading to Bcl-2 expression and chemoresistance in breast cancer cells. Mol Cancer.

[23] Ahmad A, Aboukameel A, Kong D, Wang Z, Sethi S, Chen W, Sarkar FH, Raz A (2011). Phosphoglucose isomerase/autocrine motility factor mediates epithelial–mesenchymal transition regulated by miR-200 in breast cancer cells. Cancer Res.

[24] Breving K, Esquela-Kerscher A (2010). The complexities of microRNA regulation: mirandering around the rules. Int J Biochem Cell Biol.

[25] Corcoran C, Friel AM, Duffy MJ, Crown J, O’Driscoll L (2011). Intracellular and extracellular microRNAs in breast cancer. Clin Chem.

[26] He GH, Helbing CC, Wagner MJ, Sensen CW, Riabowol K (2005). Phylogenetic analysis of the ING family of PHD finger proteins. Mol Biol Evol.

[27] Ohkouchi C, Kumamoto K, Saito M, Ishigame T, Suzuki SI, Takenoshita S, Harris CC (2017). ING2, a tumor associated gene, enhances PAI1 and HSPA1A expression with HDAC1 and mSin3A through the PHD domain and Cterminal. Mol Med Rep.

[28] Okano T, Gemma A, Hosoya Y, Hosomi Y, Nara M, Kokubo Y, Yoshimura A, Shibuya M, Nagashima M, Harris CC, Kudoh S (2006). Alterations in novel candidate tumor suppressor genes, ING1 and ING2 in human lung cancer. Oncol Rep.

[29] Zhang HK, Pan K, Wang H, Weng DS, Song HF, Zhou J, Huang W, Li JJ, Chen MS, Xia JC (2008). Decreased expression of ING2 gene and its clinicopathological significance in hepatocellular carcinoma. Cancer Lett.

[30] Walzak AA, Veldhoen N, Feng X, Riabowol K, Helbing CC (2008). Expression profiles of mRNA transcript variants encoding the human inhibitor of growth tumor suppressor gene family in normal and neoplastic tissues. Exp Cell Res.

[31] Doyon Y, Cayrou C, Ullah M, Landry AJ, Cote V, Selleck W, Lane WS, Tan S, Yang XJ, Cote J (2006). ING tumor suppressor proteins are critical regulators of chromatin acetylation required for genome expression and perpetuation. Mol Cell.

[32] Pena PV, Davrazou F, Shi X, Walter KL, Verkhusha VV, Gozani O, Zhao R, Kutateladze TG (2006). Molecular mechanism of histone H3K4me3 recognition by plant homeodomain of ING2. Nature.

[33] Shi X, Hong T, Walter KL, Ewalt M, Michishita E, Hung T, Carney D, Pena P, Lan F, Kaadige MR (2006). ING2 PHD domain links histone H3 lysine 4 methylation to active gene repression. Nature.

[34] Olaisen C, Muller R, Nedal A, Otterlei M (2015). PCNA-interacting peptides reduce Akt phosphorylation and TLR-mediated cytokine secretion suggesting a role of PCNA in cellular signaling. Cell Signal.

[35] Gong L, Li Y, Nedeljkovic-Kurepa A, Sarkar FH (2003). Inactivation of NF-kappaB by genistein is mediated via Akt signaling pathway in breast cancer cells. Oncogene.

[36] Larrieu D, Ythier D, Binet R, Brambilla C, Brambilla E, Sengupta S, Pedeux R (2009). ING2 controls the progression of DNA replication forks to maintain genome stability. EMBO Rep.

[37] Hasan S, Hassa PO, Imhof R, Hottiger MO (2001). Transcription coactivator p300 binds PCNA and may have a role in DNA repair synthesis. Nature.

[38] Vuoriluoto K, Haugen H, Kiviluoto S, Mpindi JP, Nevo J, Gjerdrum C, Tiron C, Lorens JB, Ivaska J (2011). Vimentin regulates EMT induction by Slug and oncogenic H-Ras and migration by governing Axl expression in breast cancer. Oncogene.

[39] Drago-Garcia D, Espinal-Enriquez J, Hernandez-Lemus E (2017). Network analysis of EMT and MET micro-RNA regulation in breast cancer. Sci Rep.

[40] Zavadil J, Bottinger EP (2005). TGF-beta and epithelial-to-mesenchymal transitions. Oncogene.

[41] Sarker KP, Kataoka H, Chan A, Netherton SJ, Pot I, Huynh MA, Feng X, Bonni A, Riabowol K, Bonni S (2008). ING2 as a novel mediator of transforming growth factor-beta-dependent responses in epithelial cells. J Biol Chem.

[42] Kong W, Jiang X, Mercer WE (2009). Downregulation of Wip-1 phosphatase expression in MCF-7 breast cancer cells enhances doxorubicin-induced apoptosis through p53-mediated transcriptional activation of Bax. Cancer Biol Ther.

[43] Saito M, Kumamoto K, Robles AI, Horikawa I, Furusato B, Okamura S, Goto A, Yamashita T, Nagashima M, Lee TL (2010). Targeted disruption of Ing2 results in defective spermatogenesis and development of soft-tissue sarcomas. PLoS ONE.

[44] Nagashima M, Shiseki M, Miura K, Hagiwara K, Linke SP, Pedeux R, Wang XW, Yokota J, Riabowol K, Harris CC (2001). DNA damage-inducible gene p33ING2 negatively regulates cell proliferation through acetylation of p53. Proc Natl Acad Sci USA.

